# Association Between the Implementation of Continuous Glucose Monitoring and Changes in Dietary Behavior in Insulin-Treated Patients With Diabetes Regardless of Diabetes Type

**DOI:** 10.7759/cureus.103437

**Published:** 2026-02-11

**Authors:** Daiki Arakawa, Mariko Ishisaka, Yasuji Arimura, Michikazu Nakai, Manabu Yoshimura

**Affiliations:** 1 Department of Family Medicine and Community Health, University of Miyazaki, Miyazaki, JPN; 2 Department of Health Care Research, University of Miyazaki, Miyazaki, JPN; 3 Department of Data Management, University of Miyazaki Hospital, Miyazaki, JPN; 4 Department of Family Medicine and Community Health, Tsuno Town National Health Insurance Hospital, Miyazaki, JPN

**Keywords:** continuous glucose monitoring systems, diabetes mellitus management, dietary behavior, insulin therapy diabetes, oral carbohydrate intake

## Abstract

Background: Diabetic patients receiving insulin therapy require frequent blood glucose monitoring. Recent advancements have enabled constant glucose monitoring using small sensors, such as continuous glucose monitoring (CGM) devices. Several studies have demonstrated improved HbA1c levels following CGM implementation. Glucose monitoring may influence dietary behaviors by providing real-time feedback and visualization of postprandial glucose excursions, which can enhance self-awareness and promote dietary modification. However, few studies have investigated its association with dietary behavioral changes. This study investigated the relationship between CGM implementation and changes in dietary behavior among insulin-using diabetic patients.

Methods: This study was a single-center prospective cohort study. Consecutive adult insulin-using diabetic participants were categorized into CGM and non-CGM groups based on participant intention. The primary outcome was changes in dietary behavior, defined as changes in the percentage of carbohydrates contributing to total caloric intake. Dietary intake was assessed twice at three-month intervals using the Brief-Type Self-Administered Diet History Questionnaire. A difference-in-differences analysis compared changes in carbohydrate intake percentages between the two groups.

Results: A total of 42 participants were included. The mean age was 67 ± 13 years, and the median duration of diabetes was 11 (8.0-16.0) years. Thirty-one participants used CGM, whereas 11 did not. At baseline, the mean carbohydrate intake as a percentage of total calories was 51% in the CGM group and 52% in the non-CGM group. After three months, these values were 50% and 56%, respectively. However, difference-in-differences analysis revealed no significant difference between the groups (p = 0.295).

Conclusion: CGM implementation was not significantly associated with changes in dietary behavior among insulin-using patients with diabetes. These findings indicate that CGM alone is insufficient, necessitating complementary strategies to promote dietary behavioral modification in this population.

## Introduction

In Japan, approximately 10 million individuals are estimated to have diabetes mellitus, and about 18-23% of these patients require insulin therapy [1,2]. Optimizing glycemic management in this population is therefore a major clinical priority. Self-monitoring of blood glucose is essential for achieving safe and effective glycemic control in diabetes management, particularly in insulin-treated patients [3].

In recent years, continuous glucose monitoring (CGM) devices, which measure glucose concentrations in interstitial fluid, have been introduced, providing more accurate blood glucose monitoring [4]. Although CGM use may be associated with potential downsides, such as increased cost and skin irritation, the reported benefits of CGM include safer, higher-quality glycemic control [5,6]; reduced puncture-related pain; and greater treatment satisfaction [7]. Additionally, CGM devices display blood glucose trends as a continuous line rather than as isolated values, facilitating the identification of specific patient behaviors and corresponding glycemic patterns [8]. Although exploratory, some studies suggest that CGM implementation may be associated with behavioral changes, such as increased physical activity [9]. Recently, a systematic review and meta-analysis of randomized controlled trials suggested that CGM-based feedback may function as a behavior change tool, with modest effects on health-related behaviors, although the certainty of evidence remains limited [10].

Lifestyle modification is a cornerstone of diabetes management [11], although patient adherence to such changes remains challenging [12]. CGM has been suggested to promote behavioral changes [9]; meanwhile, systematic reviews and meta-analyses of randomized controlled trials have demonstrated that other wearable devices, such as wearable activity trackers, can effectively promote physical activity [13]. However, no prospective studies have specifically investigated its impact on dietary and exercise behaviors in a primary care setting.

This prospective cohort study evaluated the relationship between CGM implementation and diet-related behavioral changes among insulin-treated diabetic patients in a primary care setting.

## Materials and methods

Study design and participants

This single-center prospective cohort study was conducted to investigate the association between the implementation of CGM and changes in dietary behavior among insulin-treated diabetic participants. The study was conducted at Takachiho National Health Insurance Hospital, the primary medical facility in Takachiho Town, Miyazaki Prefecture, Japan, serving a population of 10,000 with 120 inpatient beds. Eligible participants were patients with diabetes aged 18 years or older as of February 2023 receiving insulin therapy and performing blood glucose self-monitoring. This criterion was applied to ensure baseline comparability in glucose self-monitoring practices between the study groups. All participants provided informed consent after receiving a thorough explanation of the study.

The study population included adult patients with diabetes aged 18 years or older who were treated with insulin, including those with type 1 diabetes and type 2 diabetes. Participants receiving insulin therapy included both those treated with insulin alone and those treated with insulin in combination with oral hypoglycemic agents. There was no eligibility requirement regarding the duration of diabetes since diagnosis. Pregnant women, including those with gestational diabetes, were not included in the study population.

The exclusion criteria included the presence of other implantable medical devices (e.g., pacemakers), terminal-stage cancer, or any condition, such as cognitive impairment, that would preclude adequate understanding of the CGM device or the study procedures, as determined by the attending physician.

Research procedures

All outpatients receiving insulin therapy in 2022 were identified, and the attending physician explained CGM to those visiting the outpatient clinic between February and March 2023. Eligible participants, as determined using the inclusion and exclusion criteria, were informed that the study included individuals performing self-monitoring of blood glucose levels, and written informed consent was obtained. CGM was introduced to participants who consented and expressed a willingness to use the device. Group allocation was not randomized. After receiving standardized information about CGM, participants self-selected whether to use the device based on their personal preference. Participants who chose not to initiate CGM and continued conventional self-monitoring of blood glucose constituted the non-CGM group. The Brief-Type Self-Administered Diet History Questionnaire (BDHQ) and the International Physical Activity Questionnaire (IPAQ) were administered at baseline and three months later in both the CGM and non-CGM groups. Dietary habits were assessed using the BDHQ [14,15], whereas physical activity was evaluated with the IPAQ [16,17]. Both the BDHQ and IPAQ are validated self-administered questionnaires for use in Japanese adult populations. Participants completed the questionnaires at baseline and at three months. Dietary intake was estimated according to the BDHQ scoring algorithm, and the percentage of carbohydrates contributing to total caloric intake was calculated. Physical activity was scored according to the IPAQ scoring protocol, and total metabolic equivalent (MET) hours per week were calculated. The BDHQ is a copyrighted questionnaire; however, its use for research purposes is permitted by the developers provided that appropriate attribution is given. The IPAQ is a validated self-administered questionnaire designed to assess physical activity in adult populations and has been validated for use in Japanese adults. Physical activity was quantified using the standard IPAQ scoring protocol, and MET hours per week were calculated. CGM was performed using the FreeStyle Libre system (first-generation FreeStyle Libre, Abbott Diabetes Care, Alameda, CA, USA; approval number 22800BZX00212000) [18,19], which continuously measures subcutaneous interstitial glucose levels for 14 days using a sensor implanted in the upper arm. Participants were instructed to scan the sensor with a reader to display glucose levels from the preceding eight hours. The device was replaced by the participants themselves every two weeks. The treatment plan during the observation period remained at the discretion of the attending physician, without intervention from the study.

Measurements and outcomes

Baseline data, including age, sex, body mass index (BMI), diabetes type (type 1, type 2, and other), duration of diabetes, glycated hemoglobin (HbA1c), daily insulin dose, and other medical conditions (hypertension, dyslipidemia, coronary artery disease, and stroke), were confirmed from medical records upon study enrollment. BDHQ and IPAQ data were also collected using self-administered questionnaires. At the three-month follow-up, the HbA1c level and daily insulin dose were again confirmed from medical records, whereas the BDHQ, IPAQ, economic status, and solitary living status were assessed using follow-up questionnaires. Economic status was evaluated with a yes/no question: “Have you ever refused medical treatment recommended by a doctor for financial reasons?” Living alone status was assessed with the following question: “Do you live alone?”

The primary outcome was dietary behavior change, defined as the change in the percentage of carbohydrates in total caloric intake, as calculated using the BDHQ. Carbohydrates were selected because of their impact on postprandial blood glucose levels [20] and because low-carbohydrate diets improve glycemic control in diabetic patients [21]. Secondary outcomes included exercise behavior change, measured as MET hours per week using the IPAQ; HbA1c levels; and daily insulin dose. The number of MET hours per week was selected to measure changes in exercise behavior on the basis of physical activity standards for health promotion established by the Ministry of Health, Labour and Welfare [22].

Statistical analysis

The analysis included participants with complete baseline and three-month follow-up data. Participant characteristics were summarized using means ± standard deviations (SDs) for normally distributed continuous variables, medians (interquartile ranges, IQRs) for nonnormally distributed continuous variables, and frequencies (percentages) for categorical variables. Data normality was assessed using the Shapiro-Wilk test.

Potential confounding factors, including age, sex, economic status, and solitary living status, were identified a priori based on clinical relevance. For primary and secondary outcomes, the impact of CGM implementation was assessed using difference-in-differences (DiD) analysis. A DiD approach was used to estimate the causal effect of the intervention, comparing changes in outcomes over time between the intervention and control groups while accounting for time-invariant confounders. We further performed stratified analyses by age, sex, economic status, and solitary living status to explore potential effect modification and heterogeneity in the intervention effects across subgroups.

Only participants with complete baseline and three-month follow-up data were included in the analysis, and no imputation was performed for missing data. Statistical analysis was conducted using Stata/BE 18.0 (StataCorp, College Station, TX, USA), with significance set at p < 0.05 (two-tailed).

## Results

**Figure 1:**
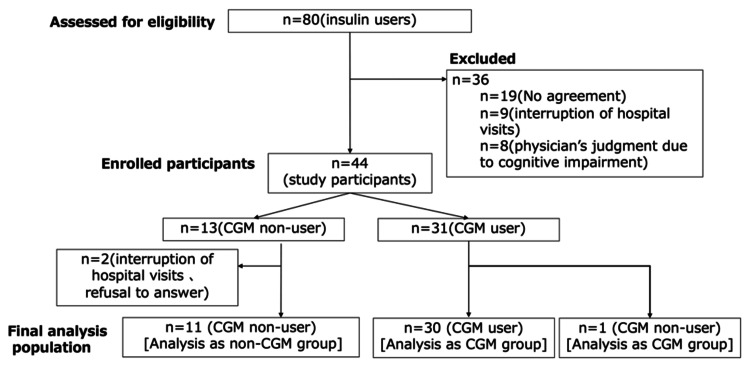
Flowchart of participant eligibility assessment, group allocation, and final analysis. Abbreviations: CGM, continuous glucose monitoring

Among the 80 insulin-using patients assessed for eligibility, 36 were excluded, and the participant selection process is summarized in Figure 1.

The remaining 44 participants were enrolled in the study. Among them, 31 chose to use CGM, whereas 13 did not. During the follow-up period, two participants in the non-CGM group discontinued hospital visits and did not complete the three-month follow-up, resulting in a final analysis population of 42 participants (31 in the CGM group and 11 in the non-CGM group). One participant in the CGM group discontinued CGM use because of personal health beliefs but completed all follow-up assessments and was included in the CGM group for analysis.

Of the 42 participants included in the final analysis, 28 were male, and 14 were female. The mean age was 67 ± 13 years, and the median duration of diabetes was 11 years. The baseline characteristics of these participants are shown in Table 1.

**Table 1:** Baseline Characteristics of the Participants Data are presented as n (%) or mean ± SD. This table is intended for descriptive purposes only; therefore, no formal statistical comparisons or test statistics are presented. Abbreviations: CGM, continuous glucose monitoring; BMI, body mass index; HbA1c, glycated hemoglobin; SD, standard deviation; IQR, interquartile range

Characteristics (%)	non-CGM group (n = 11)	CGM group (n = 31)
Age, mean±SD, year	70.3±8.2	65.8±14.0
Sex, male	5 (45)	23 (74)
BMI, median (IQR)	24.3 (23.2-28.5)	23.3 (20.5-24.8)
Type of diabetes		
Type I	1 (9)	5 (16)
Type II	9 (82)	26 (84)
Other	1 (9)	0 (0)
Duration of diabetes,median (IQR), year	10.0 (7.5-16.5)	11.0 (8.0-15.8)
HbA1c, median (IQR), %	7.3 (6.9-7.4)	7.5 (7.2-8.2)
Daily insulin dose, median (IQR), U	12.0 (8.0-31.5)	18.0 (15.0-24.5)
Other medical condition		
Hypertension	6 (55)	24 (77)
Dyslipidemia	10 (91)	20 (65)
Coronary artery disease	2 (18)	4 (13)
Stroke	1 (9)	3 (10)
Living alone		
No	7 (64)	25 (81)
Yes	4 (36)	5 (16)
Missing	0 (0)	1 (3)
No financial problem		
Yes	11 (100)	27 (87)
No	0 (0)	3 (10)
Missing	0 (0)	1 (3)

Baseline characteristics of the CGM and non-CGM groups were descriptively similar. 

Outcome

At baseline, the mean carbohydrate intake as a percentage of total calories was 51% in the CGM group and 52% in the non-CGM group. After three months, these values were 50% and 56%, respectively (Table 2).

**Table 2:** Comparison of outcomes at baseline and three months and difference-in-differences analysis Data are expressed as the mean ± standard deviation when normally distributed and as the median (interquartile range) when not. Difference-in-differences estimates were derived from linear regression models, and p-values were calculated from these models. Difference-in-differences estimates are presented only for prespecified outcomes related to dietary behavior and metabolic parameters. Total energy intake was summarized descriptively and was not included in the difference-in-differences analysis. Dietary intake was assessed using the Brief-type Self-Administered Diet History Questionnaire (BDHQ). Details regarding the questionnaire and its usage conditions are provided in the Appendix. Abbreviations: CGM, continuous glucose monitoring; MET, metabolic equivalent; HbA1c, glycated hemoglobin; NA, not applicable.

	Baseline		Three months		Difference-in-Differences Analysis	
	non-CGM group	CGM group	non-CGM group	CGM group		
n = 42	n = 11	n = 31	n = 11	n = 31	coefficient	p-value
Calorie, kcal/day	1422.1±846.7	1679.2±551.0	1394.1±703.0	1678.1±545.3	NA	NA
Carbohydrates proportion, %	52±11	51±8	56±10	50±11	-0.047	0.3
Amount of exercise,	3.30 (0-16.0)	17.03 (1.65-108.00)	6.60 (0-39.60)	26.67 (0-91.80)	-4.968	0.9
MET hours/week						
HbA1c, %	7.40 (5.50-7.10)	7.50 (7.10-8.20)	7.10 (7.00-7.25)	7.00 (6.70-7.30)	-0.059	0.86
Daily insulin dose, U	12.00 (6.00-27.00)	18.00 (15.00-25.00)	12.00 (6.00-31.50)	20.00 (14.00-25.00)	1.038	0.59

**Figure 2:**
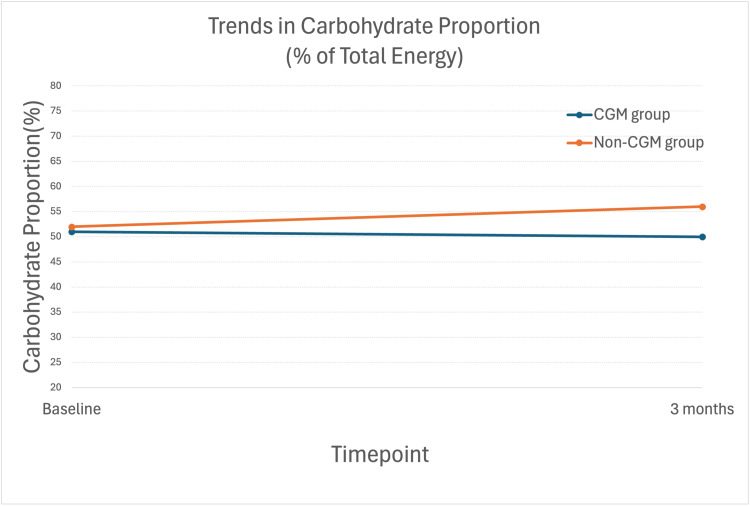
Changes in carbohydrate proportions from baseline to three months in the CGM and non-CGM groups. Abbreviations: CGM, continuous glucose monitoring

DiD analysis revealed no statistically significant difference between the groups (p = 0.295; Figure 2).

Total daily energy intake was also assessed and is summarized in Table 2; no meaningful changes or between-group differences were observed over the study period.

Similarly, no significant differences were observed in secondary outcomes, including physical activity (p = 0.908), HbA1c levels (p = 0.860), or daily insulin dose (p = 0.588) (Table 2). Stratified analyses conducted according to age, sex, economic status, and solitary living status also revealed no statistically significant differences in the primary or secondary outcomes (Table 3).

**Table 3:** Subgroup Analysis of Carbohydrate Proportions with Difference-in-Differences Analysis Data are expressed as the mean ± standard deviation. Difference-in-differences estimates were derived from linear regression models. The coefficient represents the additional change in carbohydrate proportion (%) in the CGM group compared with the non-CGM group over the observation period within each subgroup. Negative values indicate a relative decrease in carbohydrate proportion in the CGM group. P-values were calculated using linear regression models in a difference-in-differences framework. Dietary intake was assessed using the Brief-type Self-administered Diet History Questionnaire (BDHQ). Details regarding the questionnaire and its usage conditions are provided in the Appendix. Abbreviations: CGM, continuous glucose monitoring

	Baseline		Three months		Difference-in-Differences Analysis	
	Non-CGM group	CGM group	Non-CGM group	CGM group		
n = 42	n = 11	n = 31	n = 11	n = 31	coefficient	p-value
Age < 65	48±14	56±13	49±10	47±10	-0.106	0.11
Age ≥ 65	55±7	56±8	52±7	54±12	0.006	0.92
Male	51±14	55±12	50±9	50±11	-0.063	0.32
Female	54±8	57±8	53±7	52±11	-0.038	0.59
Living alone	51±13	54±11	50±8	50±11	-0.037	0.46
With family	53±9	59±7	55±8	54±13	-0.072	0.51
Not poor	52±11	56±10	51±9	50±11	-0.048	0.3

## Discussion

Summary of the results

In this prospective cohort study conducted in a primary care setting, the implementation of CGM was not associated with statistically significant changes in dietary behavior compared with usual care over a three-month period. 

Comparison with previous studies

These findings are consistent with a prior observational study by Ida et al. [9], who evaluated dietary variety, physical activity, and self-care behaviors before and after CGM implementation and reported no significant changes in dietary behavior. Unlike the earlier pre-post study without a control group, the present study incorporated a control group and still revealed no changes in dietary behavior, reinforcing the conclusion that CGM alone may not significantly influence dietary behavior.

Possible mechanisms explaining the absence of dietary behavior change despite CGM introduction are considered below, independent of the study limitations. These interpretations are speculative, as participant engagement with glucose trend data and the ability to translate such information into dietary action were not directly assessed in the present study.

First, some participants may have had limited interest in glucose trend data, which could have reduced the impact of CGM on dietary behavior [23]. Second, even among participants who were interested in glucose trends, difficulties in accessing or effectively utilizing trend data may have limited behavioral change. Notably, the CGM device used in this study lacked an alarm function, whereas newer CGM systems equipped with alert features and artificial intelligence-based glucose trend prediction tools are now available [24]. Such technologies may improve the accessibility and interpretability of glucose trend information. Third, participants may have been able to access glucose trend data appropriately but lacked the knowledge or skills required to translate this information into meaningful dietary changes. Previous studies have demonstrated an association between diabetes-related knowledge and behavioral change [25], suggesting that CGM use alone may be insufficient and that concurrent diabetes education could be necessary to promote effective lifestyle modification.

Although the association between CGM implementation and improved glycemic control has been well documented in previous studies and meta-analyses [26], the mechanisms underlying this association remain incompletely understood. Behavioral changes, as well as improved treatment adjustments by health care providers enabled by CGM data, have been proposed as potential contributors.

In this context, the present study specifically examined whether dietary behavior changes might explain the glycemic benefits associated with CGM implementation. However, no significant association between CGM use and dietary behavior change was observed. These findings suggest that factors other than dietary modification - particularly more timely or appropriate treatment adjustments by health care providers based on CGM-derived glucose profiles - may play a more important role in mediating improvements in glycemic control. Because provider-driven treatment modifications were not directly evaluated in the present study, future research with primary outcomes focusing on clinical decision-making and treatment adjustments is warranted to further elucidate the mechanisms through which CGM improves glycemic control.

Strengths and limitations of the study

This study employed a prospective cohort design with a contemporaneous control group, allowing comparison with usual care in a real-world primary care setting. Compared with simple pre-post designs, this approach provides greater methodological rigor. In addition, the use of DiD analysis enabled assessment of the association between CGM implementation and outcomes while accounting for temporal confounders that may affect both groups over time.

Several limitations should be acknowledged. First, the sample size was relatively small (n = 44), which limited the statistical power to detect modest between-group differences despite a low dropout rate (n = 2). Second, group allocation was nonrandomized and based on participant preference, introducing the potential for selection bias; although baseline characteristics were descriptively similar between groups, residual confounding cannot be excluded. Third, the three-month observation period may have been insufficient to capture sustained or gradual behavioral changes associated with CGM use, and longer follow-up is warranted.

Fourth, dietary and physical activity behaviors were assessed using self-reported questionnaires rather than objective measures such as wearable activity trackers, step counters, or direct dietary records, which may have introduced recall bias. Dietary information obtained from questionnaires may also have been influenced by recent changes in eating behavior, as assessments were conducted at discrete time points rather than through continuous monitoring. Fifth, the percentage of carbohydrate intake relative to total caloric intake was selected as the primary outcome to capture dietary behaviors relevant to glycemic control; however, there is no established consensus on a single optimal index for dietary behavior change in diabetic populations. Although alternative tools such as the Dietary Variety Score [27] and the Summary of Diabetes Self-Care Activities Measure [28] can evaluate dietary behaviors, we chose a validated questionnaire that allows quantitative assessment of macronutrient composition. Nevertheless, the absence of objective dietary measures, such as photographic food records, should be considered when interpreting the findings.

## Conclusions

In routine clinical practice, the introduction of CGM alone may not be sufficient to promote meaningful changes in dietary behavior among insulin-treated patients with diabetes. Clinicians should be aware that real-time glucose information does not automatically translate into dietary modification and may need to be complemented by structured education or behavioral support. Integrating CGM with targeted lifestyle interventions may enhance its clinical utility in primary care settings.

## Appendices

The Brief-type Self-administered Diet History Questionnaire (BDHQ) is copyrighted by the Department of Social and Preventive Epidemiology, School of Public Health, The University of Tokyo. According to the official documentation provided by the developers, the BDHQ may be used for academic research purposes without obtaining individual permission, provided that appropriate attribution is given and the questionnaire is not reproduced in full. This study complied with these conditions; therefore, the questionnaire was used for data collection but is not reproduced in this appendix.

Physical activity was assessed using the International Physical Activity Questionnaire (IPAQ), which was originally developed in English by the IPAQ Group. The questionnaire was used in accordance with the terms and conditions described by the IPAQ Group. The full questionnaire is publicly available from the official IPAQ website and is not reproduced here. Source: International Physical Activity Questionnaire (IPAQ). Available at: https://sites.google.com/view/ipaq/
 
